# Supplementary material for: Plasma Circulating mRNA Profile for the Non-Invasive Diagnosis of Colorectal Cancer Using NanoString Technologies
Source: Int J Mol Sci. 2024 Mar 5;25(5):3012. doi: 10.3390/ijms25053012 (PMC10932272; doi:10.3390/ijms25053012)
Supplement: Supplementary file 1 [file ijms-25-03012-s001.zip › Supplementary Table S1.pdf]

Supplementary Table S1. Target sequences of the CodeSet used in the customized circulating mRNA markers panel.

Gene	Accession	Position
ACTB	NM_001101.2	1011-1110

Target sequence:

TGCAGAAGGAGATCACTGCCCTGGCACCCAGCACAATGAAGATCAAGATCATTGCTCCTCCTGAGCGCAAGTACTCCGTGTGGATCGGCGGCTCCATCCT

Gene	Accession	Position
BANK1	NM_001083907.1	1396-1495

Target sequence:

GGCAAATGAAATGGAAGGGGAAGGAAAACAGAATGGATCAGGCATGGAGACCAAACACAGCCCACTAGAGGTTGGCAGTGAGAGTTCTGAAGACCAG

TAT

Gene	Accession	Position
BGN	NM_001711.3	1936-2035
**Target sequence:**		
TGTGTCTTGTGCTTCCTCAGACCTTTCTCGCTTCTGAGCTTGGTGGCCTGTTCCCTCCATCTCTCCGAACCTGGCTTCGCCTGTCCCTTTCACTCCACAC		

Gene	Accession	Position
CDA	NM_001785.2	323-422
**Target sequence:**		
AATCTTCAAAGGGTGCAACATAGAAAATGCCTGCTACCCGCTGGGCATCTGTGCTGAACGGACCGCTATCCAGAAGGCCGTCTCAGAAGGGTACAAGGAT		

Gene	Accession	Position
CEACAM7	NM_006890.4	200-299

Target sequence:

CTGCCAAACAGTGCCCAGACCAATATTGATGTCGTGCCGTTCAATGTCGCAGAAGGGAAGGAGGTCCTTCTAGTAGTCCATAATGAGTCCCAGAATCTTT

Gene	Accession	Position
CEMIP	NM_018689.1	846-945

Target sequence:

CCCAAATCAGGCACAGTCATCCATTCTGACCGGTTTGACACCTATAGATCCAAGAAAGAGAGTGAACGTCTGGTCCAGTATTTGAACGCGGTGCCCGATG

Gene	Accession	Position

CTNNB	NM_001098210.1	120-219

Target sequence:

GACGGAGGAAGGTCTGAGGAGCAGCTTCAGTCCCCGCCGAGCCGCCACCGCAGGTCGAGGACGGTCGGACTCCCGCGGCGGGAGGAGCCTGTTCCCC

TGA

Gene	Accession	Position
CTSL	NM_001912.4	1073-1172

Target sequence:

TGTGGGGCCCATTTCTGTTGCTATTGATGCAGGTCATGAGTCCTTCCTGTTCTATAAAGAAGGCATTTATTTTGAGCCAGACTGTAGCAGTGAAGACATG

Gene	Accession	Position

EPAS1	NM_001430.3	4247-4346

Target sequence:

TGCACTGAGCTATGTGACTCGGATGGTCTTTCACACGGCACATTTGGACATTTCCAGAACTACCATGAGATGGTTTAGACGGGAATTCATGCAAATGAGG

Gene	Accession	Position
FAM129C	NM_173544.4	573-672

Target sequence:

CTCAACAGCCCTGACAGGATACACGCTCCTGACTTCCCAGCGAGAATATCTCCGCCTTTTGGATGCTCTCTGCCCTGAATCCTTGGGAGACCATACTCAG

Gene	Accession	Position
FAP	NM_004460.2	1491-1590

Target sequence:

GCATTGGAAGCTATCCTCCAAGCAAGAAGTGTGTTACTTGCCATCTAAGGAAAGAAAGGTGCCAATATTACACAGCAAGTTTCAGCGACTACGCCAAGTA

Gene	Accession	Position
GK	NM_000167.3	1506-1605

Target sequence:

GGGCTGCAGAAGGAGTCGGCGTATGGAGTCTCGAACCCGAGGATTTGTCTGCCGTCACGATGGAGCGGTTTGAACCTCAGATTAATGCGGAGGAAAGTG

A

Gene	Accession	Position
GUCY2C	NM_004963.1	1841-1940

Target sequence:

CTAAGGGAATGTCATATCTGCACTCCAGTAAGACAGAAGTCCATGGTCGTCTGAAATCTACCAACTGCGTAGTGGACAGTAGAATGGTGGTGAAGATCAC

Gene	Accession	Position
INHBA	NM_002192.2	491-590

Target sequence:

GCGCTTCTGAACGCGATCAGAAAGCTTCATGTGGGCAAAGTCGGGGAGAACGGGTATGTGGAGATAGAGGATGACATTGGAAGGAGGGCAGAAATGAA
TG

Gene	Accession	Position
KRT19	NM_002276.4	139-238

Target sequence:

CGCCATGACTTCCTACAGCTATCGCCAGTCGTCGGCCACGTCGTCCTTCGGAGGCCTGGGCGGCGGCTCCGTGCGTTTTGGGCCGGGGGTCGCCTTTCGC

Gene	Accession	Position
KRT20	NM_019010.2	54-153

Target sequence:

CTCCAATGGATTTCAGTCGCAGAAGCTTCCACAGAAGCCTGAGCTCCTCCTTGCAGGCCCCTGTAGTCAGTACAGTGGGCATGCAGCGCCTCGGGACGA
C

Gene	Accession	Position
MKI67	NM_002417.2	4021-4120

Target sequence:

AGCAGATGTAGAGGGAGAACTCTTAGCGTGCAGGAATCTAATGCCATCAGCAGGCAAAGCCATGCACACGCCTAAACCATCAGTAGGTGAAGAGAAAG
AC

Gene	Accession	Position
MMP7	NM_002423.4	125-224

Target sequence:

GGGAGGCATGAGTGAGCTACAGTGGGAACAGGCTCAGGACTATCTCAAGAGATTTTATCTCTATGACTCAGAAACAAAAAATGCCAACAGTTTAGAAGCC

Gene	Accession	Position
MMP9	NM_004994.2	181-280

**Target sequence:**
CACTCGGGTGGCAGAGATGCGTGGAGAGTCGAAATCTCTGGGGCCTGCGCTGCTGCTTCTCCAGAAGCAACTGTCCCTGCCCGAGACCGGTGAGCTGGAT

Gene	Accession	Position
MS4A1	NM_152866.2	621-720
**Target sequence:**		
CTTCTGATGATCCCAGCAGGGATCTATGCACCCATCTGTGTGACTGTGTGGTACCCTCTCTGGGGAGGCATTATGTATATTATTTCCGGATCACTCCTGG		

Gene	Accession	Position
MYC	NM_002467.4	532-631
**Target sequence:**		

TTTTTTCGGGTAGTGGAAAACCAGCAGCCTCCCGCGACGATGCCCCTCAACGTTAGCTTCACCAACAGGAACTATGACCTCGACTACGACTCGGTGCAGC

Gene	Accession	Position
PIP4K2B	NM_003559.4	2691-2790

Target sequence:

ATGAGGCCCTGTGCCCTCGATCTATTTCCTTCTTCCTTCTGACCTCCTCCCAGGCACTCTTACTTCTAGCCGAACTCTTAGCTCTGGGCAGATCTCCAAG

Gene	Accession	Position
PLAUR	NM_002659.3	405-504

Target sequence:

TGGAGCTGGTGGAGAAAAGCTGTACCCACTCAGAGAAGACCAACAGGACCCTGAGCTATCGGACTGGCTTGAAGATCACCAGCCTTACCGAGGTTGTGTG

Gene	Accession	Position
PTGS2	NM_000963.3	220-319

Target sequence:

ACCGAGGTGTATGTATGAGTGTGGGATTTGACCAGTATAAGTGCGATTGTACCCGGACAGGATTCTATGGAGAAAACTGCTCAACACCGGAATTTTTGAC

Gene	Accession	Position
S100A4	NM_019554.2	379-478

Target sequence:

CGAATTCTTTGAAGGCTTCCCAGATAAGCAGCCCAGGAAGAAATGAAAACTCCTCTGATGTGGTTGGGGGGTCTGCCAGCTGGGGCCCTCCCTGTCGCCA

Gene	Accession	Position
TRIM24	NM_015905.2	541-640

Target sequence:

GCTCGCCGGTCAGCGGCTCGTCGCCGTTCGCCACCCAAGTTGGAGTCATTCGTTGCCCAGTTTGCAGCCAAGAATGTGCAGAGAGACACATCATAGATAA

Gene	Accession	Position
TUG1	NR_002323.1	2602-2701

Target sequence:

ACATCTAGGATCCCGTGAAGGTCACTGGACCCTGTTTTTTCTACTTCAAATCCTGTAGTAGCCTACTGAATGAGAAAACATATTCTGACCCATTGGGATC

Gene	Accession	Position

TYMS	NM_001071.2	356-455

Target sequence:

CTGCTGACAACCAAACGTGTGTTCTGGAAGGGTGTTTTGGAGGAGTTGCTGTGGTTTATCAAGGGATCCACAAATGCTAAAGAGCTGTCTTCCAAGGGAG
