# Supplementary material for: Plasma Circulating mRNA Profile for the Non-Invasive Diagnosis of Colorectal Cancer Using NanoString Technologies
Source: Int J Mol Sci. 2024 Mar 5;25(5):3012. doi: 10.3390/ijms25053012 (PMC10932272; doi:10.3390/ijms25053012)
Supplement: Supplementary file 1 [file ijms-25-03012-s001.zip › Supplementary Table S2.pdf]

Supplementary Table S2. Results of Mann-Whitney U test for significant differences between two groups. Differentially expressed RNAs upregulated in the plasma of CRC patients. However, no statistically significance in gene expression was observed in plasma among different stages of CRC.

**Gene Name**	**Refseq**	**Group A**	**Group A Average**	**Group B**	**Group B Average**	**p-value**	**adjusted p-value***
BANK1	NM_001083907.1	Stage I	3118.818	Stage II	3773.453	0.442617	0.484939
BANK1	NM_001083907.1	Stage I	3118.818	Stage III	3697.386	0.277144	0.41123
BANK1	NM_001083907.1	Stage I	3118.818	Stage IV	944.5667	0.082167	0.211506
BANK1	NM_001083907.1	Stage I	3118.818	Normal	169.1133	0.003381	0.019106
BANK1	NM_001083907.1	Stage I	3118.818	No stage	1436.398	0.156161	0.289552
BANK1	NM_001083907.1	Stage II	3773.453	Stage I	3118.818	0.442617	0.484939
BANK1	NM_001083907.1	Stage II	3773.453	Stage III	3697.386	0.230239	0.371082
BANK1	NM_001083907.1	Stage II	3773.453	Stage IV	944.5667	0.198033	0.338255
BANK1	NM_001083907.1	Stage II	3773.453	Normal	169.1133	0.000762	0.006719
BANK1	NM_001083907.1	Stage II	3773.453	No stage	1436.398	0.442617	0.484939
BANK1	NM_001083907.1	Stage III	3697.386	Stage I	3118.818	0.277144	0.41123
BANK1	NM_001083907.1	Stage III	3697.386	Stage II	3773.453	0.230239	0.371082
BANK1	NM_001083907.1	Stage III	3697.386	Stage IV	944.5667	0.449013	0.490644

BANK1	NM_001083907.1	Stage III	3697.386	Normal	169.1133	0.000127	0.001665
BANK1	NM_001083907.1	Stage III	3697.386	No stage	1436.398	0.248564	0.382852
BANK1	NM_001083907.1	Stage IV	944.5667	Stage I	3118.818	0.082167	0.211506
BANK1	NM_001083907.1	Stage IV	944.5667	Stage II	3773.453	0.198033	0.338255
BANK1	NM_001083907.1	Stage IV	944.5667	Stage III	3697.386	0.449013	0.490644
BANK1	NM_001083907.1	Stage IV	944.5667	Normal	169.1133	0.000621	0.005824
BANK1	NM_001083907.1	Stage IV	944.5667	No stage	1436.398	0.198033	0.338255
BANK1	NM_001083907.1	Normal	169.1133	Stage I	3118.818	0.003381	0.019106
BANK1	NM_001083907.1	Normal	169.1133	Stage II	3773.453	0.000762	0.006719
BANK1	NM_001083907.1	Normal	169.1133	Stage III	3697.386	0.000127	0.001665
BANK1	NM_001083907.1	Normal	169.1133	Stage IV	944.5667	0.000621	0.005824
BANK1	NM_001083907.1	Normal	169.1133	No stage	1436.398	0.002019	0.01298
BANK1	NM_001083907.1	No stage	1436.398	Stage I	3118.818	0.156161	0.289552
BANK1	NM_001083907.1	No stage	1436.398	Stage II	3773.453	0.442617	0.484939
BANK1	NM_001083907.1	No stage	1436.398	Stage III	3697.386	0.248564	0.382852
BANK1	NM_001083907.1	No stage	1436.398	Stage IV	944.5667	0.198033	0.338255
BANK1	NM_001083907.1	No stage	1436.398	Normal	169.1133	0.002019	0.01298
BGN	NM_001711.3	Stage I	103.0225	Stage II	191.175	0.235243	0.371082
BGN	NM_001711.3	Stage I	103.0225	Stage III	149.2763	0.464351	0.490662
BGN	NM_001711.3	Stage I	103.0225	Stage IV	123.5811	0.468372	0.490662
BGN	NM_001711.3	Stage I	103.0225	Normal	20	0.000926	0.00688
BGN	NM_001711.3	Stage I	103.0225	No stage	92.56	0.5	0.5

BGN	NM_001711.3	Stage II	191.175	Stage I	103.0225	0.235243	0.371082
BGN	NM_001711.3	Stage II	191.175	Stage III	149.2763	0.14916	0.289552
BGN	NM_001711.3	Stage II	191.175	Stage IV	123.5811	0.154567	0.289552
BGN	NM_001711.3	Stage II	191.175	Normal	20	9.06E-05	0.001384
BGN	NM_001711.3	Stage II	191.175	No stage	92.56	0.235243	0.371082
BGN	NM_001711.3	Stage III	149.2763	Stage I	103.0225	0.464351	0.490662
BGN	NM_001711.3	Stage III	149.2763	Stage II	191.175	0.14916	0.289552
BGN	NM_001711.3	Stage III	149.2763	Stage IV	123.5811	0.354855	0.455296
BGN	NM_001711.3	Stage III	149.2763	Normal	20	0.000103	0.001459
BGN	NM_001711.3	Stage III	149.2763	No stage	92.56	0.349106	0.450864
BGN	NM_001711.3	Stage IV	123.5811	Stage I	103.0225	0.468372	0.490662
BGN	NM_001711.3	Stage IV	123.5811	Stage II	191.175	0.154567	0.289552
BGN	NM_001711.3	Stage IV	123.5811	Stage III	149.2763	0.354855	0.455296
BGN	NM_001711.3	Stage IV	123.5811	Normal	20	0.002512	0.01502
BGN	NM_001711.3	Stage IV	123.5811	No stage	92.56	0.468372	0.490662
BGN	NM_001711.3	Normal	20	Stage I	103.0225	0.000926	0.00688
BGN	NM_001711.3	Normal	20	Stage II	191.175	9.06E-05	0.001384
BGN	NM_001711.3	Normal	20	Stage III	149.2763	0.000103	0.001459
BGN	NM_001711.3	Normal	20	Stage IV	123.5811	0.002512	0.01502
BGN	NM_001711.3	Normal	20	No stage	92.56	0.000926	0.00688
BGN	NM_001711.3	No stage	92.56	Stage I	103.0225	0.5	0.5
BGN	NM_001711.3	No stage	92.56	Stage II	191.175	0.235243	0.371082

BGN	NM_001711.3	No stage	92.56	Stage III	149.2763	0.349106	0.450864
BGN	NM_001711.3	No stage	92.56	Stage IV	123.5811	0.468372	0.490662
BGN	NM_001711.3	No stage	92.56	Normal	20	0.000926	0.00688
CDA	NM_001785.2	Stage I	82333	Stage II	133665.2	0.235243	0.371082
CDA	NM_001785.2	Stage I	82333	Stage III	79813.29	0.488248	0.497289
CDA	NM_001785.2	Stage I	82333	Stage IV	30877.19	0.176939	0.318722
CDA	NM_001785.2	Stage I	82333	Normal	41917.4	0.180833	0.322218
CDA	NM_001785.2	Stage I	82333	No stage	51437.12	0.235243	0.371082
CDA	NM_001785.2	Stage II	133665.2	Stage I	82333	0.235243	0.371082
CDA	NM_001785.2	Stage II	133665.2	Stage III	79813.29	0.066471	0.187162
CDA	NM_001785.2	Stage II	133665.2	Stage IV	30877.19	0.018621	0.080432
CDA	NM_001785.2	Stage II	133665.2	Normal	41917.4	0.029984	0.1195
CDA	NM_001785.2	Stage II	133665.2	No stage	51437.12	0.156161	0.289552
CDA	NM_001785.2	Stage III	79813.29	Stage I	82333	0.488248	0.497289
CDA	NM_001785.2	Stage III	79813.29	Stage II	133665.2	0.066471	0.187162
CDA	NM_001785.2	Stage III	79813.29	Stage IV	30877.19	0.009691	0.047306
CDA	NM_001785.2	Stage III	79813.29	Normal	41917.4	0.030639	0.119798
CDA	NM_001785.2	Stage III	79813.29	No stage	51437.12	0.329266	0.441731
CDA	NM_001785.2	Stage IV	30877.19	Stage I	82333	0.176939	0.318722
CDA	NM_001785.2	Stage IV	30877.19	Stage II	133665.2	0.018621	0.080432
CDA	NM_001785.2	Stage IV	30877.19	Stage III	79813.29	0.009691	0.047306
CDA	NM_001785.2	Stage IV	30877.19	Normal	41917.4	0.296776	0.427555

CDA	NM_001785.2	Stage IV	30877.19	No stage	51437.12	0.157937	0.289552
CDA	NM_001785.2	Normal	41917.4	Stage I	82333	0.180833	0.322218
CDA	NM_001785.2	Normal	41917.4	Stage II	133665.2	0.029984	0.1195
CDA	NM_001785.2	Normal	41917.4	Stage III	79813.29	0.030639	0.119798
CDA	NM_001785.2	Normal	41917.4	Stage IV	30877.19	0.296776	0.427555
CDA	NM_001785.2	Normal	41917.4	No stage	51437.12	0.215108	0.35733
CDA	NM_001785.2	No stage	51437.12	Stage I	82333	0.235243	0.371082
CDA	NM_001785.2	No stage	51437.12	Stage II	133665.2	0.156161	0.289552
CDA	NM_001785.2	No stage	51437.12	Stage III	79813.29	0.329266	0.441731
CDA	NM_001785.2	No stage	51437.12	Stage IV	30877.19	0.157937	0.289552
CDA	NM_001785.2	No stage	51437.12	Normal	41917.4	0.215108	0.35733
CEACAM7 exon1-2	NM_006890.4	Stage I	46.1025	Stage II	51.425	0.442275	0.484939
CEACAM7 exon1-2	NM_006890.4	Stage I	46.1025	Stage III	13521.77	0.361457	0.462329
CEACAM7 exon1-2	NM_006890.4	Stage I	46.1025	Stage IV	28.64333	0.110283	0.242623
CEACAM7 exon1-2	NM_006890.4	Stage I	46.1025	Normal	20	0.007667	0.038567
CEACAM7 exon1-2	NM_006890.4	Stage I	46.1025	No stage	25.2075	0.204176	0.345883
CEACAM7 exon1-2	NM_006890.4	Stage II	51.425	Stage I	46.1025	0.442275	0.484939
CEACAM7 exon1-2	NM_006890.4	Stage II	51.425	Stage III	13521.77	0.070749	0.191712
CEACAM7 exon1-2	NM_006890.4	Stage II	51.425	Stage IV	28.64333	0.026861	0.109775
CEACAM7 exon1-2	NM_006890.4	Stage II	51.425	Normal	20	9.06E-05	0.001384
CEACAM7 exon1-2	NM_006890.4	Stage II	51.425	No stage	25.2075	0.027257	0.110774
CEACAM7 exon1-2	NM_006890.4	Stage III	13521.77	Stage I	46.1025	0.361457	0.462329

CEACAM7 exon1-2	NM_006890.4	Stage III	13521.77	Stage II	51.425	0.070749	0.191712
CEACAM7 exon1-2	NM_006890.4	Stage III	13521.77	Stage IV	28.64333	0.118017	0.255549
CEACAM7 exon1-2	NM_006890.4	Stage III	13521.77	Normal	20	0.003894	0.021705
CEACAM7 exon1-2	NM_006890.4	Stage III	13521.77	No stage	25.2075	0.215264	0.35733
CEACAM7 exon1-2	NM_006890.4	Stage IV	28.64333	Stage I	46.1025	0.110283	0.242623
CEACAM7 exon1-2	NM_006890.4	Stage IV	28.64333	Stage II	51.425	0.026861	0.109775
CEACAM7 exon1-2	NM_006890.4	Stage IV	28.64333	Stage III	13521.77	0.118017	0.255549
CEACAM7 exon1-2	NM_006890.4	Stage IV	28.64333	Normal	20	0.054401	0.169761
CEACAM7 exon1-2	NM_006890.4	Stage IV	28.64333	No stage	25.2075	0.5	0.5
CEACAM7 exon1-2	NM_006890.4	Normal	20	Stage I	46.1025	0.007667	0.038567
CEACAM7 exon1-2	NM_006890.4	Normal	20	Stage II	51.425	9.06E-05	0.001384
CEACAM7 exon1-2	NM_006890.4	Normal	20	Stage III	13521.77	0.003894	0.021705
CEACAM7 exon1-2	NM_006890.4	Normal	20	Stage IV	28.64333	0.054401	0.169761
CEACAM7 exon1-2	NM_006890.4	Normal	20	No stage	25.2075	0.056176	0.169761
CEACAM7 exon1-2	NM_006890.4	No stage	25.2075	Stage I	46.1025	0.204176	0.345883
CEACAM7 exon1-2	NM_006890.4	No stage	25.2075	Stage II	51.425	0.027257	0.110774
CEACAM7 exon1-2	NM_006890.4	No stage	25.2075	Stage III	13521.77	0.215264	0.35733
CEACAM7 exon1-2	NM_006890.4	No stage	25.2075	Stage IV	28.64333	0.5	0.5
CEACAM7 exon1-2	NM_006890.4	No stage	25.2075	Normal	20	0.056176	0.169761
CEMIP	NM_018689.1	Stage I	47200.59	Stage II	59372.89	0.156161	0.289552
CEMIP	NM_018689.1	Stage I	47200.59	Stage III	23834.92	0.429596	0.484939
CEMIP	NM_018689.1	Stage I	47200.59	Stage IV	18151.22	0.319391	0.441731

CEMIP	NM_018689.1	Stage I	47200.59	Normal	4046.548	0.081739	0.211506
CEMIP	NM_018689.1	Stage I	47200.59	No stage	105721	0.5	0.5
CEMIP	NM_018689.1	Stage II	59372.89	Stage I	47200.59	0.156161	0.289552
CEMIP	NM_018689.1	Stage II	59372.89	Stage III	23834.92	0.092005	0.219168
CEMIP	NM_018689.1	Stage II	59372.89	Stage IV	18151.22	0.07024	0.191712
CEMIP	NM_018689.1	Stage II	59372.89	Normal	4046.548	0.003077	0.017509
CEMIP	NM_018689.1	Stage II	59372.89	No stage	105721	0.235243	0.371082
CEMIP	NM_018689.1	Stage III	23834.92	Stage I	47200.59	0.429596	0.484939
CEMIP	NM_018689.1	Stage III	23834.92	Stage II	59372.89	0.092005	0.219168
CEMIP	NM_018689.1	Stage III	23834.92	Stage IV	18151.22	0.405646	0.484939
CEMIP	NM_018689.1	Stage III	23834.92	Normal	4046.548	0.002077	0.01298
CEMIP	NM_018689.1	Stage III	23834.92	No stage	105721	0.452927	0.490662
CEMIP	NM_018689.1	Stage IV	18151.22	Stage I	47200.59	0.319391	0.441731
CEMIP	NM_018689.1	Stage IV	18151.22	Stage II	59372.89	0.07024	0.191712
CEMIP	NM_018689.1	Stage IV	18151.22	Stage III	23834.92	0.405646	0.484939
CEMIP	NM_018689.1	Stage IV	18151.22	Normal	4046.548	0.049078	0.164591
CEMIP	NM_018689.1	Stage IV	18151.22	No stage	105721	0.437832	0.484939
CEMIP	NM_018689.1	Normal	4046.548	Stage I	47200.59	0.081739	0.211506
CEMIP	NM_018689.1	Normal	4046.548	Stage II	59372.89	0.003077	0.017509
CEMIP	NM_018689.1	Normal	4046.548	Stage III	23834.92	0.002077	0.01298
CEMIP	NM_018689.1	Normal	4046.548	Stage IV	18151.22	0.049078	0.164591
CEMIP	NM_018689.1	Normal	4046.548	No stage	105721	0.047246	0.160405

CEMIP	NM_018689.1	No stage	105721	Stage I	47200.59	0.5	0.5
CEMIP	NM_018689.1	No stage	105721	Stage II	59372.89	0.235243	0.371082
CEMIP	NM_018689.1	No stage	105721	Stage III	23834.92	0.452927	0.490662
CEMIP	NM_018689.1	No stage	105721	Stage IV	18151.22	0.437832	0.484939
CEMIP	NM_018689.1	No stage	105721	Normal	4046.548	0.047246	0.160405
CTNNB1 exon1-2	NM_001098210.1	Stage I	15716.63	Stage II	36968.89	0.056176	0.169761
CTNNB1 exon1-2	NM_001098210.1	Stage I	15716.63	Stage III	25861.48	0.329266	0.441731
CTNNB1 exon1-2	NM_001098210.1	Stage I	15716.63	Stage IV	19006.47	0.438602	0.484939
CTNNB1 exon1-2	NM_001098210.1	Stage I	15716.63	Normal	8143.892	0.067565	0.189596
CTNNB1 exon1-2	NM_001098210.1	Stage I	15716.63	No stage	19110.33	0.442617	0.484939
CTNNB1 exon1-2	NM_001098210.1	Stage II	36968.89	Stage I	15716.63	0.056176	0.169761
CTNNB1 exon1-2	NM_001098210.1	Stage II	36968.89	Stage III	25861.48	0.083064	0.211506
CTNNB1 exon1-2	NM_001098210.1	Stage II	36968.89	Stage IV	19006.47	0.052596	0.1695
CTNNB1 exon1-2	NM_001098210.1	Stage II	36968.89	Normal	8143.892	0.00191	0.012606
CTNNB1 exon1-2	NM_001098210.1	Stage II	36968.89	No stage	19110.33	0.056176	0.169761
CTNNB1 exon1-2	NM_001098210.1	Stage III	25861.48	Stage I	15716.63	0.329266	0.441731
CTNNB1 exon1-2	NM_001098210.1	Stage III	25861.48	Stage II	36968.89	0.083064	0.211506
CTNNB1 exon1-2	NM_001098210.1	Stage III	25861.48	Stage IV	19006.47	0.12119	0.257458
CTNNB1 exon1-2	NM_001098210.1	Stage III	25861.48	Normal	8143.892	0.000382	0.003985
CTNNB1 exon1-2	NM_001098210.1	Stage III	25861.48	No stage	19110.33	0.395441	0.484939
CTNNB1 exon1-2	NM_001098210.1	Stage IV	19006.47	Stage I	15716.63	0.438602	0.484939
CTNNB1 exon1-2	NM_001098210.1	Stage IV	19006.47	Stage II	36968.89	0.052596	0.1695

CTNNB1 exon1-2	NM_001098210.1	Stage IV	19006.47	Stage III	25861.48	0.12119	0.257458
CTNNB1 exon1-2	NM_001098210.1	Stage IV	19006.47	Normal	8143.892	0.042235	0.150187
CTNNB1 exon1-2	NM_001098210.1	Stage IV	19006.47	No stage	19110.33	0.469251	0.490662
CTNNB1 exon1-2	NM_001098210.1	Normal	8143.892	Stage I	15716.63	0.067565	0.189596
CTNNB1 exon1-2	NM_001098210.1	Normal	8143.892	Stage II	36968.89	0.00191	0.012606
CTNNB1 exon1-2	NM_001098210.1	Normal	8143.892	Stage III	25861.48	0.000382	0.003985
CTNNB1 exon1-2	NM_001098210.1	Normal	8143.892	Stage IV	19006.47	0.042235	0.150187
CTNNB1 exon1-2	NM_001098210.1	Normal	8143.892	No stage	19110.33	0.037145	0.140222
CTNNB1 exon1-2	NM_001098210.1	No stage	19110.33	Stage I	15716.63	0.442617	0.484939
CTNNB1 exon1-2	NM_001098210.1	No stage	19110.33	Stage II	36968.89	0.056176	0.169761
CTNNB1 exon1-2	NM_001098210.1	No stage	19110.33	Stage III	25861.48	0.395441	0.484939
CTNNB1 exon1-2	NM_001098210.1	No stage	19110.33	Stage IV	19006.47	0.469251	0.490662
CTNNB1 exon1-2	NM_001098210.1	No stage	19110.33	Normal	8143.892	0.037145	0.140222
CTSL	NM_001912.4	Stage I	26001.42	Stage II	36041.48	0.235243	0.371082
CTSL	NM_001912.4	Stage I	26001.42	Stage III	22480.74	0.307876	0.438434
CTSL	NM_001912.4	Stage I	26001.42	Stage IV	15920.57	0.176939	0.318722
CTSL	NM_001912.4	Stage I	26001.42	Normal	3366.493	0.033209	0.129234
CTSL	NM_001912.4	Stage I	26001.42	No stage	26612.48	0.442617	0.484939
CTSL	NM_001912.4	Stage II	36041.48	Stage I	26001.42	0.235243	0.371082
CTSL	NM_001912.4	Stage II	36041.48	Stage III	22480.74	0.07421	0.196229
CTSL	NM_001912.4	Stage II	36041.48	Stage IV	15920.57	0.037991	0.140222
CTSL	NM_001912.4	Stage II	36041.48	Normal	3366.493	0.001716	0.011898

CTSL	NM_001912.4	Stage II	36041.48	No stage	26612.48	0.332503	0.441731
CTSL	NM_001912.4	Stage III	22480.74	Stage I	26001.42	0.307876	0.438434
CTSL	NM_001912.4	Stage III	22480.74	Stage II	36041.48	0.07421	0.196229
CTSL	NM_001912.4	Stage III	22480.74	Stage IV	15920.57	0.210478	0.35488
CTSL	NM_001912.4	Stage III	22480.74	Normal	3366.493	0.000747	0.006719
CTSL	NM_001912.4	Stage III	22480.74	No stage	26612.48	0.329104	0.441731
CTSL	NM_001912.4	Stage IV	15920.57	Stage I	26001.42	0.176939	0.318722
CTSL	NM_001912.4	Stage IV	15920.57	Stage II	36041.48	0.037991	0.140222
CTSL	NM_001912.4	Stage IV	15920.57	Stage III	22480.74	0.210478	0.35488
CTSL	NM_001912.4	Stage IV	15920.57	Normal	3366.493	0.01362	0.063844
CTSL	NM_001912.4	Stage IV	15920.57	No stage	26612.48	0.157937	0.289552
CTSL	NM_001912.4	Normal	3366.493	Stage I	26001.42	0.033209	0.129234
CTSL	NM_001912.4	Normal	3366.493	Stage II	36041.48	0.001716	0.011898
CTSL	NM_001912.4	Normal	3366.493	Stage III	22480.74	0.000747	0.006719
CTSL	NM_001912.4	Normal	3366.493	Stage IV	15920.57	0.01362	0.063844
CTSL	NM_001912.4	Normal	3366.493	No stage	26612.48	0.003714	0.020841
CTSL	NM_001912.4	No stage	26612.48	Stage I	26001.42	0.442617	0.484939
CTSL	NM_001912.4	No stage	26612.48	Stage II	36041.48	0.332503	0.441731
CTSL	NM_001912.4	No stage	26612.48	Stage III	22480.74	0.329104	0.441731
CTSL	NM_001912.4	No stage	26612.48	Stage IV	15920.57	0.157937	0.289552
CTSL	NM_001912.4	No stage	26612.48	Normal	3366.493	0.003714	0.020841
EPAS1	NM_001430.3	Stage I	283.21	Stage II	3203.148	0.096965	0.219168

EPAS1	NM_001430.3	Stage I	283.21	Stage III	3505.034	0.0869	0.219168
EPAS1	NM_001430.3	Stage I	283.21	Stage IV	1865.092	0.122276	0.257458
EPAS1	NM_001430.3	Stage I	283.21	Normal	1235.516	0.060794	0.178571
EPAS1	NM_001430.3	Stage I	283.21	No stage	1649.365	0.096965	0.219168
EPAS1	NM_001430.3	Stage II	3203.148	Stage I	283.21	0.096965	0.219168
EPAS1	NM_001430.3	Stage II	3203.148	Stage III	3505.034	0.350567	0.450864
EPAS1	NM_001430.3	Stage II	3203.148	Stage IV	1865.092	0.243427	0.37725
EPAS1	NM_001430.3	Stage II	3203.148	Normal	1235.516	0.010746	0.051846
EPAS1	NM_001430.3	Stage II	3203.148	No stage	1649.365	0.442617	0.484939
EPAS1	NM_001430.3	Stage III	3505.034	Stage I	283.21	0.0869	0.219168
EPAS1	NM_001430.3	Stage III	3505.034	Stage II	3203.148	0.350567	0.450864
EPAS1	NM_001430.3	Stage III	3505.034	Stage IV	1865.092	0.266539	0.400217
EPAS1	NM_001430.3	Stage III	3505.034	Normal	1235.516	0.002074	0.01298
EPAS1	NM_001430.3	Stage III	3505.034	No stage	1649.365	0.488224	0.497289
EPAS1	NM_001430.3	Stage IV	1865.092	Stage I	283.21	0.122276	0.257458
EPAS1	NM_001430.3	Stage IV	1865.092	Stage II	3203.148	0.243427	0.37725
EPAS1	NM_001430.3	Stage IV	1865.092	Stage III	3505.034	0.266539	0.400217
EPAS1	NM_001430.3	Stage IV	1865.092	Normal	1235.516	0.015165	0.068486
EPAS1	NM_001430.3	Stage IV	1865.092	No stage	1649.365	0.294321	0.426362
EPAS1	NM_001430.3	Normal	1235.516	Stage I	283.21	0.060794	0.178571
EPAS1	NM_001430.3	Normal	1235.516	Stage II	3203.148	0.010746	0.051846
EPAS1	NM_001430.3	Normal	1235.516	Stage III	3505.034	0.002074	0.01298

EPAS1	NM_001430.3	Normal	1235.516	Stage IV	1865.092	0.015165	0.068486
EPAS1	NM_001430.3	Normal	1235.516	No stage	1649.365	0.015391	0.06901
EPAS1	NM_001430.3	No stage	1649.365	Stage I	283.21	0.096965	0.219168
EPAS1	NM_001430.3	No stage	1649.365	Stage II	3203.148	0.442617	0.484939
EPAS1	NM_001430.3	No stage	1649.365	Stage III	3505.034	0.488224	0.497289
EPAS1	NM_001430.3	No stage	1649.365	Stage IV	1865.092	0.294321	0.426362
EPAS1	NM_001430.3	No stage	1649.365	Normal	1235.516	0.015391	0.06901
FAM129C	NM_173544.4	Stage I	58697.87	Stage II	99605.14	0.156161	0.289552
FAM129C	NM_173544.4	Stage I	58697.87	Stage III	59054.28	0.418302	0.484939
FAM129C	NM_173544.4	Stage I	58697.87	Stage IV	47203.52	0.321486	0.441731
FAM129C	NM_173544.4	Stage I	58697.87	Normal	16909.75	0.05612	0.169761
FAM129C	NM_173544.4	Stage I	58697.87	No stage	82975.95	0.442617	0.484939
FAM129C	NM_173544.4	Stage II	99605.14	Stage I	58697.87	0.156161	0.289552
FAM129C	NM_173544.4	Stage II	99605.14	Stage III	59054.28	0.083064	0.211506
FAM129C	NM_173544.4	Stage II	99605.14	Stage IV	47203.52	0.07134	0.191712
FAM129C	NM_173544.4	Stage II	99605.14	Normal	16909.75	0.00213	0.013213
FAM129C	NM_173544.4	Stage II	99605.14	No stage	82975.95	0.332503	0.441731
FAM129C	NM_173544.4	Stage III	59054.28	Stage I	58697.87	0.418302	0.484939
FAM129C	NM_173544.4	Stage III	59054.28	Stage II	99605.14	0.083064	0.211506
FAM129C	NM_173544.4	Stage III	59054.28	Stage IV	47203.52	0.210777	0.35488
FAM129C	NM_173544.4	Stage III	59054.28	Normal	16909.75	2.12E-05	0.001095
FAM129C	NM_173544.4	Stage III	59054.28	No stage	82975.95	0.464784	0.490662

FAM129C	NM_173544.4	Stage IV	47203.52	Stage I	58697.87	0.321486	0.441731
FAM129C	NM_173544.4	Stage IV	47203.52	Stage II	99605.14	0.07134	0.191712
FAM129C	NM_173544.4	Stage IV	47203.52	Stage III	59054.28	0.210777	0.35488
FAM129C	NM_173544.4	Stage IV	47203.52	Normal	16909.75	0.022947	0.096098
FAM129C	NM_173544.4	Stage IV	47203.52	No stage	82975.95	0.294577	0.426362
FAM129C	NM_173544.4	Normal	16909.75	Stage I	58697.87	0.05612	0.169761
FAM129C	NM_173544.4	Normal	16909.75	Stage II	99605.14	0.00213	0.013213
FAM129C	NM_173544.4	Normal	16909.75	Stage III	59054.28	2.12E-05	0.001095
FAM129C	NM_173544.4	Normal	16909.75	Stage IV	47203.52	0.022947	0.096098
FAM129C	NM_173544.4	Normal	16909.75	No stage	82975.95	0.008853	0.043475
FAM129C	NM_173544.4	No stage	82975.95	Stage I	58697.87	0.442617	0.484939
FAM129C	NM_173544.4	No stage	82975.95	Stage II	99605.14	0.332503	0.441731
FAM129C	NM_173544.4	No stage	82975.95	Stage III	59054.28	0.464784	0.490662
FAM129C	NM_173544.4	No stage	82975.95	Stage IV	47203.52	0.294577	0.426362
FAM129C	NM_173544.4	No stage	82975.95	Normal	16909.75	0.008853	0.043475
FAP	NM_004460.2	Stage I	321.02	Stage II	102.3725	0.235243	0.371082
FAP	NM_004460.2	Stage I	321.02	Stage III	869.8074	0.405272	0.484939
FAP	NM_004460.2	Stage I	321.02	Stage IV	46.50889	0.2581	0.394319
FAP	NM_004460.2	Stage I	321.02	Normal	20	0.000926	0.00688
FAP	NM_004460.2	Stage I	321.02	No stage	792.0325	0.096965	0.219168
FAP	NM_004460.2	Stage II	102.3725	Stage I	321.02	0.235243	0.371082
FAP	NM_004460.2	Stage II	102.3725	Stage III	869.8074	0.488103	0.497289

FAP	NM_004460.2	Stage II	102.3725	Stage IV	46.50889	0.065791	0.187162
FAP	NM_004460.2	Stage II	102.3725	Normal	20	9.06E-05	0.001384
FAP	NM_004460.2	Stage II	102.3725	No stage	792.0325	0.015191	0.068486
FAP	NM_004460.2	Stage III	869.8074	Stage I	321.02	0.405272	0.484939
FAP	NM_004460.2	Stage III	869.8074	Stage II	102.3725	0.488103	0.497289
FAP	NM_004460.2	Stage III	869.8074	Stage IV	46.50889	0.054775	0.169761
FAP	NM_004460.2	Stage III	869.8074	Normal	20	0.000185	0.00228
FAP	NM_004460.2	Stage III	869.8074	No stage	792.0325	0.071944	0.192707
FAP	NM_004460.2	Stage IV	46.50889	Stage I	321.02	0.2581	0.394319
FAP	NM_004460.2	Stage IV	46.50889	Stage II	102.3725	0.065791	0.187162
FAP	NM_004460.2	Stage IV	46.50889	Stage III	869.8074	0.054775	0.169761
FAP	NM_004460.2	Stage IV	46.50889	Normal	20	0.007431	0.038558
FAP	NM_004460.2	Stage IV	46.50889	No stage	792.0325	0.002737	0.01613
FAP	NM_004460.2	Normal	20	Stage I	321.02	0.000926	0.00688
FAP	NM_004460.2	Normal	20	Stage II	102.3725	9.06E-05	0.001384
FAP	NM_004460.2	Normal	20	Stage III	869.8074	0.000185	0.00228
FAP	NM_004460.2	Normal	20	Stage IV	46.50889	0.007431	0.038558
FAP	NM_004460.2	Normal	20	No stage	792.0325	9.06E-05	0.001384
FAP	NM_004460.2	No stage	792.0325	Stage I	321.02	0.096965	0.219168
FAP	NM_004460.2	No stage	792.0325	Stage II	102.3725	0.015191	0.068486
FAP	NM_004460.2	No stage	792.0325	Stage III	869.8074	0.071944	0.192707
FAP	NM_004460.2	No stage	792.0325	Stage IV	46.50889	0.002737	0.01613

FAP	NM_004460.2	No stage	792.0325	Normal	20	9.06E-05	0.001384
GK	NM_000167.3	Stage I	19534.83	Stage II	29865.46	0.235243	0.371082
GK	NM_000167.3	Stage I	19534.83	Stage III	20696.59	0.488243	0.497289
GK	NM_000167.3	Stage I	19534.83	Stage IV	10550.17	0.378649	0.47475
GK	NM_000167.3	Stage I	19534.83	Normal	3633.967	0.062177	0.179986
GK	NM_000167.3	Stage I	19534.83	No stage	17962.45	0.442617	0.484939
GK	NM_000167.3	Stage II	29865.46	Stage I	19534.83	0.235243	0.371082
GK	NM_000167.3	Stage II	29865.46	Stage III	20696.59	0.165433	0.301286
GK	NM_000167.3	Stage II	29865.46	Stage IV	10550.17	0.07134	0.191712
GK	NM_000167.3	Stage II	29865.46	Normal	3633.967	0.00828	0.041399
GK	NM_000167.3	Stage II	29865.46	No stage	17962.45	0.156161	0.289552
GK	NM_000167.3	Stage III	20696.59	Stage I	19534.83	0.488243	0.497289
GK	NM_000167.3	Stage III	20696.59	Stage II	29865.46	0.165433	0.301286
GK	NM_000167.3	Stage III	20696.59	Stage IV	10550.17	0.180473	0.322218
GK	NM_000167.3	Stage III	20696.59	Normal	3633.967	0.000671	0.006223
GK	NM_000167.3	Stage III	20696.59	No stage	17962.45	0.488247	0.497289
GK	NM_000167.3	Stage IV	10550.17	Stage I	19534.83	0.378649	0.47475
GK	NM_000167.3	Stage IV	10550.17	Stage II	29865.46	0.07134	0.191712
GK	NM_000167.3	Stage IV	10550.17	Stage III	20696.59	0.180473	0.322218
GK	NM_000167.3	Stage IV	10550.17	Normal	3633.967	0.011996	0.057537
GK	NM_000167.3	Stage IV	10550.17	No stage	17962.45	0.408481	0.484939
GK	NM_000167.3	Normal	3633.967	Stage I	19534.83	0.062177	0.179986

GK	NM_000167.3	Normal	3633.967	Stage II	29865.46	0.00828	0.041399
GK	NM_000167.3	Normal	3633.967	Stage III	20696.59	0.000671	0.006223
GK	NM_000167.3	Normal	3633.967	Stage IV	10550.17	0.011996	0.057537
GK	NM_000167.3	Normal	3633.967	No stage	17962.45	0.068162	0.190624
GK	NM_000167.3	No stage	17962.45	Stage I	19534.83	0.442617	0.484939
GK	NM_000167.3	No stage	17962.45	Stage II	29865.46	0.156161	0.289552
GK	NM_000167.3	No stage	17962.45	Stage III	20696.59	0.488247	0.497289
GK	NM_000167.3	No stage	17962.45	Stage IV	10550.17	0.408481	0.484939
GK	NM_000167.3	No stage	17962.45	Normal	3633.967	0.068162	0.190624
GUCY2C	NM_004963.1	Stage I	61.775	Stage II	97.025	0.096965	0.219168
GUCY2C	NM_004963.1	Stage I	61.775	Stage III	494.8544	0.394183	0.484939
GUCY2C	NM_004963.1	Stage I	61.775	Stage IV	68.26778	0.468372	0.490662
GUCY2C	NM_004963.1	Stage I	61.775	Normal	20	0.000926	0.00688
GUCY2C	NM_004963.1	Stage I	61.775	No stage	120.17	0.015191	0.068486
GUCY2C	NM_004963.1	Stage II	97.025	Stage I	61.775	0.096965	0.219168
GUCY2C	NM_004963.1	Stage II	97.025	Stage III	494.8544	0.14916	0.289552
GUCY2C	NM_004963.1	Stage II	97.025	Stage IV	68.26778	0.194737	0.338255
GUCY2C	NM_004963.1	Stage II	97.025	Normal	20	9.06E-05	0.001384
GUCY2C	NM_004963.1	Stage II	97.025	No stage	120.17	0.156161	0.289552
GUCY2C	NM_004963.1	Stage III	494.8544	Stage I	61.775	0.394183	0.484939
GUCY2C	NM_004963.1	Stage III	494.8544	Stage II	97.025	0.14916	0.289552
GUCY2C	NM_004963.1	Stage III	494.8544	Stage IV	68.26778	0.341097	0.449529

GUCY2C	NM_004963.1	Stage III	494.8544	Normal	20	0.000103	0.001459
GUCY2C	NM_004963.1	Stage III	494.8544	No stage	120.17	0.051093	0.168605
GUCY2C	NM_004963.1	Stage IV	68.26778	Stage I	61.775	0.468372	0.490662
GUCY2C	NM_004963.1	Stage IV	68.26778	Stage II	97.025	0.194737	0.338255
GUCY2C	NM_004963.1	Stage IV	68.26778	Stage III	494.8544	0.341097	0.449529
GUCY2C	NM_004963.1	Stage IV	68.26778	Normal	20	0.002512	0.01502
GUCY2C	NM_004963.1	Stage IV	68.26778	No stage	120.17	0.068581	0.191147
GUCY2C	NM_004963.1	Normal	20	Stage I	61.775	0.000926	0.00688
GUCY2C	NM_004963.1	Normal	20	Stage II	97.025	9.06E-05	0.001384
GUCY2C	NM_004963.1	Normal	20	Stage III	494.8544	0.000103	0.001459
GUCY2C	NM_004963.1	Normal	20	Stage IV	68.26778	0.002512	0.01502
GUCY2C	NM_004963.1	Normal	20	No stage	120.17	9.06E-05	0.001384
GUCY2C	NM_004963.1	No stage	120.17	Stage I	61.775	0.015191	0.068486
GUCY2C	NM_004963.1	No stage	120.17	Stage II	97.025	0.156161	0.289552
GUCY2C	NM_004963.1	No stage	120.17	Stage III	494.8544	0.051093	0.168605
GUCY2C	NM_004963.1	No stage	120.17	Stage IV	68.26778	0.068581	0.191147
GUCY2C	NM_004963.1	No stage	120.17	Normal	20	9.06E-05	0.001384
INHBA	NM_002192.2	Stage I	50134.49	Stage II	86119.04	0.156161	0.289552
INHBA	NM_002192.2	Stage I	50134.49	Stage III	54501	0.372885	0.47115
INHBA	NM_002192.2	Stage I	50134.49	Stage IV	47530.9	0.438602	0.484939
INHBA	NM_002192.2	Stage I	50134.49	Normal	18310.55	0.048283	0.162585
INHBA	NM_002192.2	Stage I	50134.49	No stage	53943.73	0.442617	0.484939

INHBA	NM_002192.2	Stage II	86119.04	Stage I	50134.49	0.156161	0.289552
INHBA	NM_002192.2	Stage II	86119.04	Stage III	54501	0.066451	0.187162
INHBA	NM_002192.2	Stage II	86119.04	Stage IV	47530.9	0.037991	0.140222
INHBA	NM_002192.2	Stage II	86119.04	Normal	18310.55	0.002991	0.017253
INHBA	NM_002192.2	Stage II	86119.04	No stage	53943.73	0.096965	0.219168
INHBA	NM_002192.2	Stage III	54501	Stage I	50134.49	0.372885	0.47115
INHBA	NM_002192.2	Stage III	54501	Stage II	86119.04	0.066451	0.187162
INHBA	NM_002192.2	Stage III	54501	Stage IV	47530.9	0.456352	0.490662
INHBA	NM_002192.2	Stage III	54501	Normal	18310.55	0.000422	0.004353
INHBA	NM_002192.2	Stage III	54501	No stage	53943.73	0.418294	0.484939
INHBA	NM_002192.2	Stage IV	47530.9	Stage I	50134.49	0.438602	0.484939
INHBA	NM_002192.2	Stage IV	47530.9	Stage II	86119.04	0.037991	0.140222
INHBA	NM_002192.2	Stage IV	47530.9	Stage III	54501	0.456352	0.490662
INHBA	NM_002192.2	Stage IV	47530.9	Normal	18310.55	0.020741	0.08912
INHBA	NM_002192.2	Stage IV	47530.9	No stage	53943.73	0.469251	0.490662
INHBA	NM_002192.2	Normal	18310.55	Stage I	50134.49	0.048283	0.162585
INHBA	NM_002192.2	Normal	18310.55	Stage II	86119.04	0.002991	0.017253
INHBA	NM_002192.2	Normal	18310.55	Stage III	54501	0.000422	0.004353
INHBA	NM_002192.2	Normal	18310.55	Stage IV	47530.9	0.020741	0.08912
INHBA	NM_002192.2	Normal	18310.55	No stage	53943.73	0.004312	0.023873
INHBA	NM_002192.2	No stage	53943.73	Stage I	50134.49	0.442617	0.484939
INHBA	NM_002192.2	No stage	53943.73	Stage II	86119.04	0.096965	0.219168

INHBA	NM_002192.2	No stage	53943.73	Stage III	54501	0.418294	0.484939
INHBA	NM_002192.2	No stage	53943.73	Stage IV	47530.9	0.469251	0.490662
INHBA	NM_002192.2	No stage	53943.73	Normal	18310.55	0.004312	0.023873
KRT19 exon1-2	NM_002276.4	Stage I	12402.46	Stage II	32193.63	0.156161	0.289552
KRT19 exon1-2	NM_002276.4	Stage I	12402.46	Stage III	26178.84	0.125268	0.258365
KRT19 exon1-2	NM_002276.4	Stage I	12402.46	Stage IV	29384.17	0.157937	0.289552
KRT19 exon1-2	NM_002276.4	Stage I	12402.46	Normal	4029.44	0.047246	0.160405
KRT19 exon1-2	NM_002276.4	Stage I	12402.46	No stage	17119.72	0.332503	0.441731
KRT19 exon1-2	NM_002276.4	Stage II	32193.63	Stage I	12402.46	0.156161	0.289552
KRT19 exon1-2	NM_002276.4	Stage II	32193.63	Stage III	26178.84	0.151224	0.289552
KRT19 exon1-2	NM_002276.4	Stage II	32193.63	Stage IV	29384.17	0.243727	0.37725
KRT19 exon1-2	NM_002276.4	Stage II	32193.63	Normal	4029.44	0.003077	0.017509
KRT19 exon1-2	NM_002276.4	Stage II	32193.63	No stage	17119.72	0.096965	0.219168
KRT19 exon1-2	NM_002276.4	Stage III	26178.84	Stage I	12402.46	0.125268	0.258365
KRT19 exon1-2	NM_002276.4	Stage III	26178.84	Stage II	32193.63	0.151224	0.289552
KRT19 exon1-2	NM_002276.4	Stage III	26178.84	Stage IV	29384.17	0.255399	0.391643
KRT19 exon1-2	NM_002276.4	Stage III	26178.84	Normal	4029.44	3.67E-05	0.001384
KRT19 exon1-2	NM_002276.4	Stage III	26178.84	No stage	17119.72	0.248999	0.382852
KRT19 exon1-2	NM_002276.4	Stage IV	29384.17	Stage I	12402.46	0.157937	0.289552
KRT19 exon1-2	NM_002276.4	Stage IV	29384.17	Stage II	32193.63	0.243727	0.37725
KRT19 exon1-2	NM_002276.4	Stage IV	29384.17	Stage III	26178.84	0.255399	0.391643
KRT19 exon1-2	NM_002276.4	Stage IV	29384.17	Normal	4029.44	0.000495	0.005016

KRT19 exon1-2	NM_002276.4	Stage IV	29384.17	No stage	17119.72	0.469251	0.490662
KRT19 exon1-2	NM_002276.4	Normal	4029.44	Stage I	12402.46	0.047246	0.160405
KRT19 exon1-2	NM_002276.4	Normal	4029.44	Stage II	32193.63	0.003077	0.017509
KRT19 exon1-2	NM_002276.4	Normal	4029.44	Stage III	26178.84	3.67E-05	0.001384
KRT19 exon1-2	NM_002276.4	Normal	4029.44	Stage IV	29384.17	0.000495	0.005016
KRT19 exon1-2	NM_002276.4	Normal	4029.44	No stage	17119.72	0.006715	0.035972
KRT19 exon1-2	NM_002276.4	No stage	17119.72	Stage I	12402.46	0.332503	0.441731
KRT19 exon1-2	NM_002276.4	No stage	17119.72	Stage II	32193.63	0.096965	0.219168
KRT19 exon1-2	NM_002276.4	No stage	17119.72	Stage III	26178.84	0.248999	0.382852
KRT19 exon1-2	NM_002276.4	No stage	17119.72	Stage IV	29384.17	0.469251	0.490662
KRT19 exon1-2	NM_002276.4	No stage	17119.72	Normal	4029.44	0.006715	0.035972
KRT20 exon1-2	NM_019010.2	Stage I	46344.5	Stage II	116612.9	0.056176	0.169761
KRT20 exon1-2	NM_019010.2	Stage I	46344.5	Stage III	78925.36	0.28781	0.422497
KRT20 exon1-2	NM_019010.2	Stage I	46344.5	Stage IV	56187.71	0.438602	0.484939
KRT20 exon1-2	NM_019010.2	Stage I	46344.5	Normal	10225.4	0.048283	0.162585
KRT20 exon1-2	NM_019010.2	Stage I	46344.5	No stage	80124.04	0.235243	0.371082
KRT20 exon1-2	NM_019010.2	Stage II	116612.9	Stage I	46344.5	0.056176	0.169761
KRT20 exon1-2	NM_019010.2	Stage II	116612.9	Stage III	78925.36	0.046539	0.159314
KRT20 exon1-2	NM_019010.2	Stage II	116612.9	Stage IV	56187.71	0.052596	0.1695
KRT20 exon1-2	NM_019010.2	Stage II	116612.9	Normal	10225.4	0.002046	0.01298
KRT20 exon1-2	NM_019010.2	Stage II	116612.9	No stage	80124.04	0.156161	0.289552
KRT20 exon1-2	NM_019010.2	Stage III	78925.36	Stage I	46344.5	0.28781	0.422497

KRT20 exon1-2	NM_019010.2	Stage III	78925.36	Stage II	116612.9	0.046539	0.159314
KRT20 exon1-2	NM_019010.2	Stage III	78925.36	Stage IV	56187.71	0.16197	0.295631
KRT20 exon1-2	NM_019010.2	Stage III	78925.36	Normal	10225.4	0.000137	0.00177
KRT20 exon1-2	NM_019010.2	Stage III	78925.36	No stage	80124.04	0.350854	0.450864
KRT20 exon1-2	NM_019010.2	Stage IV	56187.71	Stage I	46344.5	0.438602	0.484939
KRT20 exon1-2	NM_019010.2	Stage IV	56187.71	Stage II	116612.9	0.052596	0.1695
KRT20 exon1-2	NM_019010.2	Stage IV	56187.71	Stage III	78925.36	0.16197	0.295631
KRT20 exon1-2	NM_019010.2	Stage IV	56187.71	Normal	10225.4	0.024583	0.101913
KRT20 exon1-2	NM_019010.2	Stage IV	56187.71	No stage	80124.04	0.12358	0.257458
KRT20 exon1-2	NM_019010.2	Normal	10225.4	Stage I	46344.5	0.048283	0.162585
KRT20 exon1-2	NM_019010.2	Normal	10225.4	Stage II	116612.9	0.002046	0.01298
KRT20 exon1-2	NM_019010.2	Normal	10225.4	Stage III	78925.36	0.000137	0.00177
KRT20 exon1-2	NM_019010.2	Normal	10225.4	Stage IV	56187.71	0.024583	0.101913
KRT20 exon1-2	NM_019010.2	Normal	10225.4	No stage	80124.04	0.002046	0.01298
KRT20 exon1-2	NM_019010.2	No stage	80124.04	Stage I	46344.5	0.235243	0.371082
KRT20 exon1-2	NM_019010.2	No stage	80124.04	Stage II	116612.9	0.156161	0.289552
KRT20 exon1-2	NM_019010.2	No stage	80124.04	Stage III	78925.36	0.350854	0.450864
KRT20 exon1-2	NM_019010.2	No stage	80124.04	Stage IV	56187.71	0.12358	0.257458
KRT20 exon1-2	NM_019010.2	No stage	80124.04	Normal	10225.4	0.002046	0.01298
MKI67	NM_002417.2	Stage I	56987.24	Stage II	74004.38	0.332503	0.441731
MKI67	NM_002417.2	Stage I	56987.24	Stage III	77026.36	0.308232	0.438434
MKI67	NM_002417.2	Stage I	56987.24	Stage IV	52881.03	0.438602	0.484939

MKI67	NM_002417.2	Stage I	56987.24	Normal	22054.26	0.078453	0.206786
MKI67	NM_002417.2	Stage I	56987.24	No stage	47471.99	0.442617	0.484939
MKI67	NM_002417.2	Stage II	74004.38	Stage I	56987.24	0.332503	0.441731
MKI67	NM_002417.2	Stage II	74004.38	Stage III	77026.36	0.125268	0.258365
MKI67	NM_002417.2	Stage II	74004.38	Stage IV	52881.03	0.198033	0.338255
MKI67	NM_002417.2	Stage II	74004.38	Normal	22054.26	0.002991	0.017253
MKI67	NM_002417.2	Stage II	74004.38	No stage	47471.99	0.096965	0.219168
MKI67	NM_002417.2	Stage III	77026.36	Stage I	56987.24	0.308232	0.438434
MKI67	NM_002417.2	Stage III	77026.36	Stage II	74004.38	0.125268	0.258365
MKI67	NM_002417.2	Stage III	77026.36	Stage IV	52881.03	0.221483	0.363796
MKI67	NM_002417.2	Stage III	77026.36	Normal	22054.26	0.001822	0.012458
MKI67	NM_002417.2	Stage III	77026.36	No stage	47471.99	0.418302	0.484939
MKI67	NM_002417.2	Stage IV	52881.03	Stage I	56987.24	0.438602	0.484939
MKI67	NM_002417.2	Stage IV	52881.03	Stage II	74004.38	0.198033	0.338255
MKI67	NM_002417.2	Stage IV	52881.03	Stage III	77026.36	0.221483	0.363796
MKI67	NM_002417.2	Stage IV	52881.03	Normal	22054.26	0.105317	0.232939
MKI67	NM_002417.2	Stage IV	52881.03	No stage	47471.99	0.408481	0.484939
MKI67	NM_002417.2	Normal	22054.26	Stage I	56987.24	0.078453	0.206786
MKI67	NM_002417.2	Normal	22054.26	Stage II	74004.38	0.002991	0.017253
MKI67	NM_002417.2	Normal	22054.26	Stage III	77026.36	0.001822	0.012458
MKI67	NM_002417.2	Normal	22054.26	Stage IV	52881.03	0.105317	0.232939
MKI67	NM_002417.2	Normal	22054.26	No stage	47471.99	0.01626	0.071734

MKI67	NM_002417.2	No stage	47471.99	Stage I	56987.24	0.442617	0.484939
MKI67	NM_002417.2	No stage	47471.99	Stage II	74004.38	0.096965	0.219168
MKI67	NM_002417.2	No stage	47471.99	Stage III	77026.36	0.418302	0.484939
MKI67	NM_002417.2	No stage	47471.99	Stage IV	52881.03	0.408481	0.484939
MKI67	NM_002417.2	No stage	47471.99	Normal	22054.26	0.01626	0.071734
MMP7 exon1-2	NM_002423.4	Stage I	3649.235	Stage II	277.8825	0.442617	0.484939
MMP7 exon1-2	NM_002423.4	Stage I	3649.235	Stage III	382.3	0.476422	0.492626
MMP7 exon1-2	NM_002423.4	Stage I	3649.235	Stage IV	120.2722	0.377165	0.47475
MMP7 exon1-2	NM_002423.4	Stage I	3649.235	Normal	20	0.000926	0.00688
MMP7 exon1-2	NM_002423.4	Stage I	3649.235	No stage	147.9875	0.442617	0.484939
MMP7 exon1-2	NM_002423.4	Stage II	277.8825	Stage I	3649.235	0.442617	0.484939
MMP7 exon1-2	NM_002423.4	Stage II	277.8825	Stage III	382.3	0.441325	0.484939
MMP7 exon1-2	NM_002423.4	Stage II	277.8825	Stage IV	120.2722	0.1566	0.289552
MMP7 exon1-2	NM_002423.4	Stage II	277.8825	Normal	20	9.06E-05	0.001384
MMP7 exon1-2	NM_002423.4	Stage II	277.8825	No stage	147.9875	0.442617	0.484939
MMP7 exon1-2	NM_002423.4	Stage III	382.3	Stage I	3649.235	0.476422	0.492626
MMP7 exon1-2	NM_002423.4	Stage III	382.3	Stage II	277.8825	0.441325	0.484939
MMP7 exon1-2	NM_002423.4	Stage III	382.3	Stage IV	120.2722	0.131286	0.26943
MMP7 exon1-2	NM_002423.4	Stage III	382.3	Normal	20	1.47E-05	0.000916
MMP7 exon1-2	NM_002423.4	Stage III	382.3	No stage	147.9875	0.307876	0.438434
MMP7 exon1-2	NM_002423.4	Stage IV	120.2722	Stage I	3649.235	0.377165	0.47475
MMP7 exon1-2	NM_002423.4	Stage IV	120.2722	Stage II	277.8825	0.1566	0.289552

MMP7 exon1-2	NM_002423.4	Stage IV	120.2722	Stage III	382.3	0.131286	0.26943
MMP7 exon1-2	NM_002423.4	Stage IV	120.2722	Normal	20	0.000782	0.006719
MMP7 exon1-2	NM_002423.4	Stage IV	120.2722	No stage	147.9875	0.242522	0.37725
MMP7 exon1-2	NM_002423.4	Normal	20	Stage I	3649.235	0.000926	0.00688
MMP7 exon1-2	NM_002423.4	Normal	20	Stage II	277.8825	9.06E-05	0.001384
MMP7 exon1-2	NM_002423.4	Normal	20	Stage III	382.3	1.47E-05	0.000916
MMP7 exon1-2	NM_002423.4	Normal	20	Stage IV	120.2722	0.000782	0.006719
MMP7 exon1-2	NM_002423.4	Normal	20	No stage	147.9875	9.06E-05	0.001384
MMP7 exon1-2	NM_002423.4	No stage	147.9875	Stage I	3649.235	0.442617	0.484939
MMP7 exon1-2	NM_002423.4	No stage	147.9875	Stage II	277.8825	0.442617	0.484939
MMP7 exon1-2	NM_002423.4	No stage	147.9875	Stage III	382.3	0.307876	0.438434
MMP7 exon1-2	NM_002423.4	No stage	147.9875	Stage IV	120.2722	0.242522	0.37725
MMP7 exon1-2	NM_002423.4	No stage	147.9875	Normal	20	9.06E-05	0.001384
MMP9 exon1-2	NM_004994.2	Stage I	367.665	Stage II	6548.393	0.156161	0.289552
MMP9 exon1-2	NM_004994.2	Stage I	367.665	Stage III	7647.711	0.230239	0.371082
MMP9 exon1-2	NM_004994.2	Stage I	367.665	Stage IV	3684.182	0.5	0.5
MMP9 exon1-2	NM_004994.2	Stage I	367.665	Normal	20	0.000926	0.00688
MMP9 exon1-2	NM_004994.2	Stage I	367.665	No stage	232.725	0.385752	0.478564
MMP9 exon1-2	NM_004994.2	Stage II	6548.393	Stage I	367.665	0.156161	0.289552
MMP9 exon1-2	NM_004994.2	Stage II	6548.393	Stage III	7647.711	0.287618	0.422497
MMP9 exon1-2	NM_004994.2	Stage II	6548.393	Stage IV	3684.182	0.1566	0.289552
MMP9 exon1-2	NM_004994.2	Stage II	6548.393	Normal	20	9.06E-05	0.001384

MMP9 exon1-2	NM_004994.2	Stage II	6548.393	No stage	232.725	0.056176	0.169761
MMP9 exon1-2	NM_004994.2	Stage III	7647.711	Stage I	367.665	0.230239	0.371082
MMP9 exon1-2	NM_004994.2	Stage III	7647.711	Stage II	6548.393	0.287618	0.422497
MMP9 exon1-2	NM_004994.2	Stage III	7647.711	Stage IV	3684.182	0.231685	0.371082
MMP9 exon1-2	NM_004994.2	Stage III	7647.711	Normal	20	7.26E-06	0.000916
MMP9 exon1-2	NM_004994.2	Stage III	7647.711	No stage	232.725	0.092005	0.219168
MMP9 exon1-2	NM_004994.2	Stage IV	3684.182	Stage I	367.665	0.5	0.5
MMP9 exon1-2	NM_004994.2	Stage IV	3684.182	Stage II	6548.393	0.1566	0.289552
MMP9 exon1-2	NM_004994.2	Stage IV	3684.182	Stage III	7647.711	0.231685	0.371082
MMP9 exon1-2	NM_004994.2	Stage IV	3684.182	Normal	20	0.000782	0.006719
MMP9 exon1-2	NM_004994.2	Stage IV	3684.182	No stage	232.725	0.319391	0.441731
MMP9 exon1-2	NM_004994.2	Normal	20	Stage I	367.665	0.000926	0.00688
MMP9 exon1-2	NM_004994.2	Normal	20	Stage II	6548.393	9.06E-05	0.001384
MMP9 exon1-2	NM_004994.2	Normal	20	Stage III	7647.711	7.26E-06	0.000916
MMP9 exon1-2	NM_004994.2	Normal	20	Stage IV	3684.182	0.000782	0.006719
MMP9 exon1-2	NM_004994.2	Normal	20	No stage	232.725	0.000926	0.00688
MMP9 exon1-2	NM_004994.2	No stage	232.725	Stage I	367.665	0.385752	0.478564
MMP9 exon1-2	NM_004994.2	No stage	232.725	Stage II	6548.393	0.056176	0.169761
MMP9 exon1-2	NM_004994.2	No stage	232.725	Stage III	7647.711	0.092005	0.219168
MMP9 exon1-2	NM_004994.2	No stage	232.725	Stage IV	3684.182	0.319391	0.441731
MMP9 exon1-2	NM_004994.2	No stage	232.725	Normal	20	0.000926	0.00688
MS4A1	NM_152866.2	Stage I	18589.93	Stage II	22468.3	0.332503	0.441731

MS4A1	NM_152866.2	Stage I	18589.93	Stage III	17110.81	0.5	0.5
MS4A1	NM_152866.2	Stage I	18589.93	Stage IV	7012.719	0.268267	0.400217
MS4A1	NM_152866.2	Stage I	18589.93	Normal	650.3958	0.007491	0.038567
MS4A1	NM_152866.2	Stage I	18589.93	No stage	12686.44	0.442617	0.484939
MS4A1	NM_152866.2	Stage II	22468.3	Stage I	18589.93	0.332503	0.441731
MS4A1	NM_152866.2	Stage II	22468.3	Stage III	17110.81	0.09236	0.219168
MS4A1	NM_152866.2	Stage II	22468.3	Stage IV	7012.719	0.026878	0.109775
MS4A1	NM_152866.2	Stage II	22468.3	Normal	650.3958	0.000529	0.005016
MS4A1	NM_152866.2	Stage II	22468.3	No stage	12686.44	0.156161	0.289552
MS4A1	NM_152866.2	Stage III	17110.81	Stage I	18589.93	0.5	0.5
MS4A1	NM_152866.2	Stage III	17110.81	Stage II	22468.3	0.09236	0.219168
MS4A1	NM_152866.2	Stage III	17110.81	Stage IV	7012.719	0.069364	0.191712
MS4A1	NM_152866.2	Stage III	17110.81	Normal	650.3958	1.52E-05	0.000916
MS4A1	NM_152866.2	Stage III	17110.81	No stage	12686.44	0.418269	0.484939
MS4A1	NM_152866.2	Stage IV	7012.719	Stage I	18589.93	0.268267	0.400217
MS4A1	NM_152866.2	Stage IV	7012.719	Stage II	22468.3	0.026878	0.109775
MS4A1	NM_152866.2	Stage IV	7012.719	Stage III	17110.81	0.069364	0.191712
MS4A1	NM_152866.2	Stage IV	7012.719	Normal	650.3958	0.002541	0.015082
MS4A1	NM_152866.2	Stage IV	7012.719	No stage	12686.44	0.07134	0.191712
MS4A1	NM_152866.2	Normal	650.3958	Stage I	18589.93	0.007491	0.038567
MS4A1	NM_152866.2	Normal	650.3958	Stage II	22468.3	0.000529	0.005016
MS4A1	NM_152866.2	Normal	650.3958	Stage III	17110.81	1.52E-05	0.000916

MS4A1	NM_152866.2	Normal	650.3958	Stage IV	7012.719	0.002541	0.015082
MS4A1	NM_152866.2	Normal	650.3958	No stage	12686.44	0.000858	0.00688
MS4A1	NM_152866.2	No stage	12686.44	Stage I	18589.93	0.442617	0.484939
MS4A1	NM_152866.2	No stage	12686.44	Stage II	22468.3	0.156161	0.289552
MS4A1	NM_152866.2	No stage	12686.44	Stage III	17110.81	0.418269	0.484939
MS4A1	NM_152866.2	No stage	12686.44	Stage IV	7012.719	0.07134	0.191712
MS4A1	NM_152866.2	No stage	12686.44	Normal	650.3958	0.000858	0.00688
MYC exon1-2	NM_002467.4	Stage I	13616.51	Stage II	34941.22	0.030301	0.119609
MYC exon1-2	NM_002467.4	Stage I	13616.51	Stage III	27283.9	0.221806	0.363796
MYC exon1-2	NM_002467.4	Stage I	13616.51	Stage IV	26276.21	0.438602	0.484939
MYC exon1-2	NM_002467.4	Stage I	13616.51	Normal	5521.895	0.056779	0.170957
MYC exon1-2	NM_002467.4	Stage I	13616.51	No stage	15963.85	0.442617	0.484939
MYC exon1-2	NM_002467.4	Stage II	34941.22	Stage I	13616.51	0.030301	0.119609
MYC exon1-2	NM_002467.4	Stage II	34941.22	Stage III	27283.9	0.102596	0.22876
MYC exon1-2	NM_002467.4	Stage II	34941.22	Stage IV	26276.21	0.094831	0.219168
MYC exon1-2	NM_002467.4	Stage II	34941.22	Normal	5521.895	0.001716	0.011898
MYC exon1-2	NM_002467.4	Stage II	34941.22	No stage	15963.85	0.096965	0.219168
MYC exon1-2	NM_002467.4	Stage III	27283.9	Stage I	13616.51	0.221806	0.363796
MYC exon1-2	NM_002467.4	Stage III	27283.9	Stage II	34941.22	0.102596	0.22876
MYC exon1-2	NM_002467.4	Stage III	27283.9	Stage IV	26276.21	0.157515	0.289552
MYC exon1-2	NM_002467.4	Stage III	27283.9	Normal	5521.895	0.000267	0.00282
MYC exon1-2	NM_002467.4	Stage III	27283.9	No stage	15963.85	0.28781	0.422497

MYC exon1-2	NM_002467.4	Stage IV	26276.21	Stage I	13616.51	0.438602	0.484939
MYC exon1-2	NM_002467.4	Stage IV	26276.21	Stage II	34941.22	0.094831	0.219168
MYC exon1-2	NM_002467.4	Stage IV	26276.21	Stage III	27283.9	0.157515	0.289552
MYC exon1-2	NM_002467.4	Stage IV	26276.21	Normal	5521.895	0.038057	0.140222
MYC exon1-2	NM_002467.4	Stage IV	26276.21	No stage	15963.85	0.469251	0.490662
MYC exon1-2	NM_002467.4	Normal	5521.895	Stage I	13616.51	0.056779	0.170957
MYC exon1-2	NM_002467.4	Normal	5521.895	Stage II	34941.22	0.001716	0.011898
MYC exon1-2	NM_002467.4	Normal	5521.895	Stage III	27283.9	0.000267	0.00282
MYC exon1-2	NM_002467.4	Normal	5521.895	Stage IV	26276.21	0.038057	0.140222
MYC exon1-2	NM_002467.4	Normal	5521.895	No stage	15963.85	0.035501	0.136862
MYC exon1-2	NM_002467.4	No stage	15963.85	Stage I	13616.51	0.442617	0.484939
MYC exon1-2	NM_002467.4	No stage	15963.85	Stage II	34941.22	0.096965	0.219168
MYC exon1-2	NM_002467.4	No stage	15963.85	Stage III	27283.9	0.28781	0.422497
MYC exon1-2	NM_002467.4	No stage	15963.85	Stage IV	26276.21	0.469251	0.490662
MYC exon1-2	NM_002467.4	No stage	15963.85	Normal	5521.895	0.035501	0.136862
PIP4K2B	NM_003559.4	Stage I	308.945	Stage II	6175.323	0.156161	0.289552
PIP4K2B	NM_003559.4	Stage I	308.945	Stage III	1391.916	0.476105	0.492626
PIP4K2B	NM_003559.4	Stage I	308.945	Stage IV	1687.758	0.372739	0.47115
PIP4K2B	NM_003559.4	Stage I	308.945	Normal	20	0.000926	0.00688
PIP4K2B	NM_003559.4	Stage I	308.945	No stage	287.83	0.44121	0.484939
PIP4K2B	NM_003559.4	Stage II	6175.323	Stage I	308.945	0.156161	0.289552
PIP4K2B	NM_003559.4	Stage II	6175.323	Stage III	1391.916	0.064115	0.184304

PIP4K2B	NM_003559.4	Stage II	6175.323	Stage IV	1687.758	0.088642	0.219168
PIP4K2B	NM_003559.4	Stage II	6175.323	Normal	20	9.06E-05	0.001384
PIP4K2B	NM_003559.4	Stage II	6175.323	No stage	287.83	0.154712	0.289552
PIP4K2B	NM_003559.4	Stage III	1391.916	Stage I	308.945	0.476105	0.492626
PIP4K2B	NM_003559.4	Stage III	1391.916	Stage II	6175.323	0.064115	0.184304
PIP4K2B	NM_003559.4	Stage III	1391.916	Stage IV	1687.758	0.243038	0.37725
PIP4K2B	NM_003559.4	Stage III	1391.916	Normal	20	0.000185	0.00228
PIP4K2B	NM_003559.4	Stage III	1391.916	No stage	287.83	0.370123	0.47115
PIP4K2B	NM_003559.4	Stage IV	1687.758	Stage I	308.945	0.372739	0.47115
PIP4K2B	NM_003559.4	Stage IV	1687.758	Stage II	6175.323	0.088642	0.219168
PIP4K2B	NM_003559.4	Stage IV	1687.758	Stage III	1391.916	0.243038	0.37725
PIP4K2B	NM_003559.4	Stage IV	1687.758	Normal	20	0.007431	0.038558
PIP4K2B	NM_003559.4	Stage IV	1687.758	No stage	287.83	0.337475	0.446897
PIP4K2B	NM_003559.4	Normal	20	Stage I	308.945	0.000926	0.00688
PIP4K2B	NM_003559.4	Normal	20	Stage II	6175.323	9.06E-05	0.001384
PIP4K2B	NM_003559.4	Normal	20	Stage III	1391.916	0.000185	0.00228
PIP4K2B	NM_003559.4	Normal	20	Stage IV	1687.758	0.007431	0.038558
PIP4K2B	NM_003559.4	Normal	20	No stage	287.83	0.007667	0.038567
PIP4K2B	NM_003559.4	No stage	287.83	Stage I	308.945	0.44121	0.484939
PIP4K2B	NM_003559.4	No stage	287.83	Stage II	6175.323	0.154712	0.289552
PIP4K2B	NM_003559.4	No stage	287.83	Stage III	1391.916	0.370123	0.47115
PIP4K2B	NM_003559.4	No stage	287.83	Stage IV	1687.758	0.337475	0.446897

PIP4K2B	NM_003559.4	No stage	287.83	Normal	20	0.007667	0.038567
PLAUR exon2-3	NM_002659.3	Stage I	565.765	Stage II	12562.74	0.015191	0.068486
PLAUR exon2-3	NM_002659.3	Stage I	565.765	Stage III	6378.578	0.062086	0.179986
PLAUR exon2-3	NM_002659.3	Stage I	565.765	Stage IV	9516.01	0.196727	0.338255
PLAUR exon2-3	NM_002659.3	Stage I	565.765	Normal	1035.063	0.060794	0.178571
PLAUR exon2-3	NM_002659.3	Stage I	565.765	No stage	3826.875	0.030301	0.119609
PLAUR exon2-3	NM_002659.3	Stage II	12562.74	Stage I	565.765	0.015191	0.068486
PLAUR exon2-3	NM_002659.3	Stage II	12562.74	Stage III	6378.578	0.082637	0.211506
PLAUR exon2-3	NM_002659.3	Stage II	12562.74	Stage IV	9516.01	0.157604	0.289552
PLAUR exon2-3	NM_002659.3	Stage II	12562.74	Normal	1035.063	0.001368	0.0099
PLAUR exon2-3	NM_002659.3	Stage II	12562.74	No stage	3826.875	0.096965	0.219168
PLAUR exon2-3	NM_002659.3	Stage III	6378.578	Stage I	565.765	0.062086	0.179986
PLAUR exon2-3	NM_002659.3	Stage III	6378.578	Stage II	12562.74	0.082637	0.211506
PLAUR exon2-3	NM_002659.3	Stage III	6378.578	Stage IV	9516.01	0.441699	0.484939
PLAUR exon2-3	NM_002659.3	Stage III	6378.578	Normal	1035.063	0.000736	0.006719
PLAUR exon2-3	NM_002659.3	Stage III	6378.578	No stage	3826.875	0.488224	0.497289
PLAUR exon2-3	NM_002659.3	Stage IV	9516.01	Stage I	565.765	0.196727	0.338255
PLAUR exon2-3	NM_002659.3	Stage IV	9516.01	Stage II	12562.74	0.157604	0.289552
PLAUR exon2-3	NM_002659.3	Stage IV	9516.01	Stage III	6378.578	0.441699	0.484939
PLAUR exon2-3	NM_002659.3	Stage IV	9516.01	Normal	1035.063	0.006555	0.035344
PLAUR exon2-3	NM_002659.3	Stage IV	9516.01	No stage	3826.875	0.349641	0.450864
PLAUR exon2-3	NM_002659.3	Normal	1035.063	Stage I	565.765	0.060794	0.178571

PLAUR exon2-3	NM_002659.3	Normal	1035.063	Stage II	12562.74	0.001368	0.0099
PLAUR exon2-3	NM_002659.3	Normal	1035.063	Stage III	6378.578	0.000736	0.006719
PLAUR exon2-3	NM_002659.3	Normal	1035.063	Stage IV	9516.01	0.006555	0.035344
PLAUR exon2-3	NM_002659.3	Normal	1035.063	No stage	3826.875	0.007373	0.038558
PLAUR exon2-3	NM_002659.3	No stage	3826.875	Stage I	565.765	0.030301	0.119609
PLAUR exon2-3	NM_002659.3	No stage	3826.875	Stage II	12562.74	0.096965	0.219168
PLAUR exon2-3	NM_002659.3	No stage	3826.875	Stage III	6378.578	0.488224	0.497289
PLAUR exon2-3	NM_002659.3	No stage	3826.875	Stage IV	9516.01	0.349641	0.450864
PLAUR exon2-3	NM_002659.3	No stage	3826.875	Normal	1035.063	0.007373	0.038558
PTGS2 exon2-3	NM_000963.3	Stage I	27295.66	Stage II	20618.94	0.331559	0.441731
PTGS2 exon2-3	NM_000963.3	Stage I	27295.66	Stage III	25963.25	0.440109	0.484939
PTGS2 exon2-3	NM_000963.3	Stage I	27295.66	Stage IV	16800.94	0.466579	0.490662
PTGS2 exon2-3	NM_000963.3	Stage I	27295.66	Normal	20	0.007667	0.038567
PTGS2 exon2-3	NM_000963.3	Stage I	27295.66	No stage	4420.32	0.438891	0.484939
PTGS2 exon2-3	NM_000963.3	Stage II	20618.94	Stage I	27295.66	0.331559	0.441731
PTGS2 exon2-3	NM_000963.3	Stage II	20618.94	Stage III	25963.25	0.122373	0.257458
PTGS2 exon2-3	NM_000963.3	Stage II	20618.94	Stage IV	16800.94	0.116936	0.253875
PTGS2 exon2-3	NM_000963.3	Stage II	20618.94	Normal	20	9.06E-05	0.001384
PTGS2 exon2-3	NM_000963.3	Stage II	20618.94	No stage	4420.32	0.154712	0.289552
PTGS2 exon2-3	NM_000963.3	Stage III	25963.25	Stage I	27295.66	0.440109	0.484939
PTGS2 exon2-3	NM_000963.3	Stage III	25963.25	Stage II	20618.94	0.122373	0.257458
PTGS2 exon2-3	NM_000963.3	Stage III	25963.25	Stage IV	16800.94	0.318945	0.441731

PTGS2 exon2-3	NM_000963.3	Stage III	25963.25	Normal	20	0.000185	0.00228
PTGS2 exon2-3	NM_000963.3	Stage III	25963.25	No stage	4420.32	0.3042	0.437221
PTGS2 exon2-3	NM_000963.3	Stage IV	16800.94	Stage I	27295.66	0.466579	0.490662
PTGS2 exon2-3	NM_000963.3	Stage IV	16800.94	Stage II	20618.94	0.116936	0.253875
PTGS2 exon2-3	NM_000963.3	Stage IV	16800.94	Stage III	25963.25	0.318945	0.441731
PTGS2 exon2-3	NM_000963.3	Stage IV	16800.94	Normal	20	0.007431	0.038558
PTGS2 exon2-3	NM_000963.3	Stage IV	16800.94	No stage	4420.32	0.466579	0.490662
PTGS2 exon2-3	NM_000963.3	Normal	20	Stage I	27295.66	0.007667	0.038567
PTGS2 exon2-3	NM_000963.3	Normal	20	Stage II	20618.94	9.06E-05	0.001384
PTGS2 exon2-3	NM_000963.3	Normal	20	Stage III	25963.25	0.000185	0.00228
PTGS2 exon2-3	NM_000963.3	Normal	20	Stage IV	16800.94	0.007431	0.038558
PTGS2 exon2-3	NM_000963.3	Normal	20	No stage	4420.32	0.007667	0.038567
PTGS2 exon2-3	NM_000963.3	No stage	4420.32	Stage I	27295.66	0.438891	0.484939
PTGS2 exon2-3	NM_000963.3	No stage	4420.32	Stage II	20618.94	0.154712	0.289552
PTGS2 exon2-3	NM_000963.3	No stage	4420.32	Stage III	25963.25	0.3042	0.437221
PTGS2 exon2-3	NM_000963.3	No stage	4420.32	Stage IV	16800.94	0.466579	0.490662
PTGS2 exon2-3	NM_000963.3	No stage	4420.32	Normal	20	0.007667	0.038567
S100A4 exon3-4	NM_019554.2	Stage I	33689	Stage II	44015.95	0.332503	0.441731
S100A4 exon3-4	NM_019554.2	Stage I	33689	Stage III	37234.61	0.464784	0.490662
S100A4 exon3-4	NM_019554.2	Stage I	33689	Stage IV	25325.74	0.198033	0.338255
S100A4 exon3-4	NM_019554.2	Stage I	33689	Normal	18203.82	0.07132	0.191712
S100A4 exon3-4	NM_019554.2	Stage I	33689	No stage	26682.23	0.235243	0.371082

S100A4 exon3-4	NM_019554.2	Stage II	44015.95	Stage I	33689	0.332503	0.441731
S100A4 exon3-4	NM_019554.2	Stage II	44015.95	Stage III	37234.61	0.092449	0.219168
S100A4 exon3-4	NM_019554.2	Stage II	44015.95	Stage IV	25325.74	0.037991	0.140222
S100A4 exon3-4	NM_019554.2	Stage II	44015.95	Normal	18203.82	0.006327	0.034343
S100A4 exon3-4	NM_019554.2	Stage II	44015.95	No stage	26682.23	0.056176	0.169761
S100A4 exon3-4	NM_019554.2	Stage III	37234.61	Stage I	33689	0.464784	0.490662
S100A4 exon3-4	NM_019554.2	Stage III	37234.61	Stage II	44015.95	0.092449	0.219168
S100A4 exon3-4	NM_019554.2	Stage III	37234.61	Stage IV	25325.74	0.03975	0.143206
S100A4 exon3-4	NM_019554.2	Stage III	37234.61	Normal	18203.82	0.001827	0.012458
S100A4 exon3-4	NM_019554.2	Stage III	37234.61	No stage	26682.23	0.18053	0.322218
S100A4 exon3-4	NM_019554.2	Stage IV	25325.74	Stage I	33689	0.198033	0.338255
S100A4 exon3-4	NM_019554.2	Stage IV	25325.74	Stage II	44015.95	0.037991	0.140222
S100A4 exon3-4	NM_019554.2	Stage IV	25325.74	Stage III	37234.61	0.03975	0.143206
S100A4 exon3-4	NM_019554.2	Stage IV	25325.74	Normal	18203.82	0.296776	0.427555
S100A4 exon3-4	NM_019554.2	Stage IV	25325.74	No stage	26682.23	0.469251	0.490662
S100A4 exon3-4	NM_019554.2	Normal	18203.82	Stage I	33689	0.07132	0.191712
S100A4 exon3-4	NM_019554.2	Normal	18203.82	Stage II	44015.95	0.006327	0.034343
S100A4 exon3-4	NM_019554.2	Normal	18203.82	Stage III	37234.61	0.001827	0.012458
S100A4 exon3-4	NM_019554.2	Normal	18203.82	Stage IV	25325.74	0.296776	0.427555
S100A4 exon3-4	NM_019554.2	Normal	18203.82	No stage	26682.23	0.214598	0.35733
S100A4 exon3-4	NM_019554.2	No stage	26682.23	Stage I	33689	0.235243	0.371082
S100A4 exon3-4	NM_019554.2	No stage	26682.23	Stage II	44015.95	0.056176	0.169761

S100A4 exon3-4	NM_019554.2	No stage	26682.23	Stage III	37234.61	0.18053	0.322218
S100A4 exon3-4	NM_019554.2	No stage	26682.23	Stage IV	25325.74	0.469251	0.490662
S100A4 exon3-4	NM_019554.2	No stage	26682.23	Normal	18203.82	0.214598	0.35733
TRIM24	NM_015905.2	Stage I	592.65	Stage II	8514.685	0.156161	0.289552
TRIM24	NM_015905.2	Stage I	592.65	Stage III	7197.833	0.041017	0.146654
TRIM24	NM_015905.2	Stage I	592.65	Stage IV	3787.37	0.044596	0.156561
TRIM24	NM_015905.2	Stage I	592.65	Normal	20	0.000926	0.00688
TRIM24	NM_015905.2	Stage I	592.65	No stage	456.5675	0.332503	0.441731
TRIM24	NM_015905.2	Stage II	8514.685	Stage I	592.65	0.156161	0.289552
TRIM24	NM_015905.2	Stage II	8514.685	Stage III	7197.833	0.372922	0.47115
TRIM24	NM_015905.2	Stage II	8514.685	Stage IV	3787.37	0.469251	0.490662
TRIM24	NM_015905.2	Stage II	8514.685	Normal	20	9.06E-05	0.001384
TRIM24	NM_015905.2	Stage II	8514.685	No stage	456.5675	0.156161	0.289552
TRIM24	NM_015905.2	Stage III	7197.833	Stage I	592.65	0.041017	0.146654
TRIM24	NM_015905.2	Stage III	7197.833	Stage II	8514.685	0.372922	0.47115
TRIM24	NM_015905.2	Stage III	7197.833	Stage IV	3787.37	0.343854	0.450864
TRIM24	NM_015905.2	Stage III	7197.833	Normal	20	1.60E-06	0.000916
TRIM24	NM_015905.2	Stage III	7197.833	No stage	456.5675	0.041063	0.146654
TRIM24	NM_015905.2	Stage IV	3787.37	Stage I	592.65	0.044596	0.156561
TRIM24	NM_015905.2	Stage IV	3787.37	Stage II	8514.685	0.469251	0.490662
TRIM24	NM_015905.2	Stage IV	3787.37	Stage III	7197.833	0.343854	0.450864
TRIM24	NM_015905.2	Stage IV	3787.37	Normal	20	5.60E-05	0.001384

TRIM24	NM_015905.2	Stage IV	3787.37	No stage	456.5675	0.026878	0.109775
TRIM24	NM_015905.2	Normal	20	Stage I	592.65	0.000926	0.00688
TRIM24	NM_015905.2	Normal	20	Stage II	8514.685	9.06E-05	0.001384
TRIM24	NM_015905.2	Normal	20	Stage III	7197.833	1.60E-06	0.000916
TRIM24	NM_015905.2	Normal	20	Stage IV	3787.37	5.60E-05	0.001384
TRIM24	NM_015905.2	Normal	20	No stage	456.5675	9.06E-05	0.001384
TRIM24	NM_015905.2	No stage	456.5675	Stage I	592.65	0.332503	0.441731
TRIM24	NM_015905.2	No stage	456.5675	Stage II	8514.685	0.156161	0.289552
TRIM24	NM_015905.2	No stage	456.5675	Stage III	7197.833	0.041063	0.146654
TRIM24	NM_015905.2	No stage	456.5675	Stage IV	3787.37	0.026878	0.109775
TRIM24	NM_015905.2	No stage	456.5675	Normal	20	9.06E-05	0.001384
TUG1	NR_002323.1	Stage I	975.32	Stage II	5631.128	0.056176	0.169761
TUG1	NR_002323.1	Stage I	975.32	Stage III	8363.993	0.195958	0.338255
TUG1	NR_002323.1	Stage I	975.32	Stage IV	21629.69	0.108206	0.238689
TUG1	NR_002323.1	Stage I	975.32	Normal	2689.192	0.060794	0.178571
TUG1	NR_002323.1	Stage I	975.32	No stage	493.98	0.385752	0.478564
TUG1	NR_002323.1	Stage II	5631.128	Stage I	975.32	0.056176	0.169761
TUG1	NR_002323.1	Stage II	5631.128	Stage III	8363.993	0.212929	0.35733
TUG1	NR_002323.1	Stage II	5631.128	Stage IV	21629.69	0.349838	0.450864
TUG1	NR_002323.1	Stage II	5631.128	Normal	2689.192	0.010746	0.051846
TUG1	NR_002323.1	Stage II	5631.128	No stage	493.98	0.056176	0.169761
TUG1	NR_002323.1	Stage III	8363.993	Stage I	975.32	0.195958	0.338255

TUG1	NR_002323.1	Stage III	8363.993	Stage II	5631.128	0.212929	0.35733
TUG1	NR_002323.1	Stage III	8363.993	Stage IV	21629.69	0.279179	0.412764
TUG1	NR_002323.1	Stage III	8363.993	Normal	2689.192	0.001192	0.008783
TUG1	NR_002323.1	Stage III	8363.993	No stage	493.98	0.113059	0.247576
TUG1	NR_002323.1	Stage IV	21629.69	Stage I	975.32	0.108206	0.238689
TUG1	NR_002323.1	Stage IV	21629.69	Stage II	5631.128	0.349838	0.450864
TUG1	NR_002323.1	Stage IV	21629.69	Stage III	8363.993	0.279179	0.412764
TUG1	NR_002323.1	Stage IV	21629.69	Normal	2689.192	0.005063	0.027847
TUG1	NR_002323.1	Stage IV	21629.69	No stage	493.98	0.061154	0.178908
TUG1	NR_002323.1	Normal	2689.192	Stage I	975.32	0.060794	0.178571
TUG1	NR_002323.1	Normal	2689.192	Stage II	5631.128	0.010746	0.051846
TUG1	NR_002323.1	Normal	2689.192	Stage III	8363.993	0.001192	0.008783
TUG1	NR_002323.1	Normal	2689.192	Stage IV	21629.69	0.005063	0.027847
TUG1	NR_002323.1	Normal	2689.192	No stage	493.98	0.060794	0.178571
TUG1	NR_002323.1	No stage	493.98	Stage I	975.32	0.385752	0.478564
TUG1	NR_002323.1	No stage	493.98	Stage II	5631.128	0.056176	0.169761
TUG1	NR_002323.1	No stage	493.98	Stage III	8363.993	0.113059	0.247576
TUG1	NR_002323.1	No stage	493.98	Stage IV	21629.69	0.061154	0.178908
TUG1	NR_002323.1	No stage	493.98	Normal	2689.192	0.060794	0.178571
TYMS exon2-3	NM_001071.2	Stage I	30818.32	Stage II	50162.11	0.235243	0.371082
TYMS exon2-3	NM_001071.2	Stage I	30818.32	Stage III	39236.93	0.418302	0.484939
TYMS exon2-3	NM_001071.2	Stage I	30818.32	Stage IV	33363.06	0.378649	0.47475

TYMS exon2-3	NM_001071.2	Stage I	30818.32	Normal	13571.37	0.098099	0.221124
TYMS exon2-3	NM_001071.2	Stage I	30818.32	No stage	32978.77	0.442617	0.484939
TYMS exon2-3	NM_001071.2	Stage II	50162.11	Stage I	30818.32	0.235243	0.371082
TYMS exon2-3	NM_001071.2	Stage II	50162.11	Stage III	39236.93	0.213163	0.35733
TYMS exon2-3	NM_001071.2	Stage II	50162.11	Stage IV	33363.06	0.198033	0.338255
TYMS exon2-3	NM_001071.2	Stage II	50162.11	Normal	13571.37	0.038242	0.140222
TYMS exon2-3	NM_001071.2	Stage II	50162.11	No stage	32978.77	0.235243	0.371082
TYMS exon2-3	NM_001071.2	Stage III	39236.93	Stage I	30818.32	0.418302	0.484939
TYMS exon2-3	NM_001071.2	Stage III	39236.93	Stage II	50162.11	0.213163	0.35733
TYMS exon2-3	NM_001071.2	Stage III	39236.93	Stage IV	33363.06	0.088232	0.219168
TYMS exon2-3	NM_001071.2	Stage III	39236.93	Normal	13571.37	0.000219	0.002568
TYMS exon2-3	NM_001071.2	Stage III	39236.93	No stage	32978.77	0.464784	0.490662
TYMS exon2-3	NM_001071.2	Stage IV	33363.06	Stage I	30818.32	0.378649	0.47475
TYMS exon2-3	NM_001071.2	Stage IV	33363.06	Stage II	50162.11	0.198033	0.338255
TYMS exon2-3	NM_001071.2	Stage IV	33363.06	Stage III	39236.93	0.088232	0.219168
TYMS exon2-3	NM_001071.2	Stage IV	33363.06	Normal	13571.37	0.105317	0.232939
TYMS exon2-3	NM_001071.2	Stage IV	33363.06	No stage	32978.77	0.198033	0.338255
TYMS exon2-3	NM_001071.2	Normal	13571.37	Stage I	30818.32	0.098099	0.221124
TYMS exon2-3	NM_001071.2	Normal	13571.37	Stage II	50162.11	0.038242	0.140222
TYMS exon2-3	NM_001071.2	Normal	13571.37	Stage III	39236.93	0.000219	0.002568
TYMS exon2-3	NM_001071.2	Normal	13571.37	Stage IV	33363.06	0.105317	0.232939
TYMS exon2-3	NM_001071.2	Normal	13571.37	No stage	32978.77	0.01626	0.071734

TYMS exon2-3	NM_001071.2	No stage	32978.77	Stage I	30818.32	0.442617	0.484939
TYMS exon2-3	NM_001071.2	No stage	32978.77	Stage II	50162.11	0.235243	0.371082
TYMS exon2-3	NM_001071.2	No stage	32978.77	Stage III	39236.93	0.464784	0.490662
TYMS exon2-3	NM_001071.2	No stage	32978.77	Stage IV	33363.06	0.198033	0.338255
TYMS exon2-3	NM_001071.2	No stage	32978.77	Normal	13571.37	0.01626	0.071734

*adjusted p-value using Benjamini-Hochberg procedure
